# Lead-free organic–inorganic azetidinium alternating metal cation bromide: [(CH_2_)_3_NH_2_]_2_AgBiBr_6_, a perovskite-related absorber†

**DOI:** 10.1039/d3ra05966a

**Published:** 2023-12-12

**Authors:** Young Un Jin, Bernd Marler, Andrei D. Karabanov, Kristina Winkler, Ian Chang Jie Yap, Astita Dubey, Leon Spee, Marianela Escobar Castillo, Franziska Muckel, Andrei N. Salak, Niels Benson, Doru C. Lupascu

**Affiliations:** a Institute for Materials Science, Center for Nanointegration Duisburg-Essen (CENIDE), University of Duisburg-Essen 45141 Essen Germany doru.lupascu@uni-due.de; b Institute of Geology, Mineralogy and Geophysics, Ruhr-University Bochum 44780 Bochum Germany; c Institute of Technology for Nanostructures (NST), University of Duisburg-Essen 47057 Duisburg Germany; d Fraunhofer Institute for Solar Energy Systems (ISE) 79110 Freiburg Germany; e Electroenergetic Functional Materials (EEFM), CENIDE, University Duisburg-Essen 47057 Duisburg Germany; f Werkstoffe der Elektrotechnik (WET), CENIDE, University Duisburg-Essen 47057 Duisburg Germany; g Department of Materials and Ceramics Engineering, CICECO-Aveiro Institute of Materials, University of Aveiro 3810-193 Aveiro Portugal

## Abstract

In the last decade, organic–inorganic hybrid halide perovskite materials have developed into a very large research area in photovoltaics and optoelectronics as promising light harvesters. Lead-free double perovskites have recently been investigated as an environmentally friendly alternative to the lead-containing compositions. However, lead-free organic–inorganic hybrid halide double perovskites have so far rarely been produced due to a certain complexity in their synthesis. A number of small molecular cations have been investigated, but compositions containing azetidinium, which is a 4-membered heterocyclic molecular ring, on the A-site have hardly been considered. This study investigates the potential of [(CH_2_)_3_NH_2_]_2_AgBiBr_6_ as an optical absorber in photovoltaics or optoelectronics. The use of this alternative cation changes the crystal symmetry significantly. Columns of alternating metal cation form which are separated by the organic ions. While crystal symmetry is rather different from the perovskites, the overall properties as an absorber are similar. It is thus worthwhile to further investigate alternate hybrid compositions which form into other symmetries than the perovskite base structure.

## Abstract 2

A new metal bromide perovskite derivative, azetidinium silver bismuth bromide is synthesized as a light-absorbing material.

## Graphical abstract

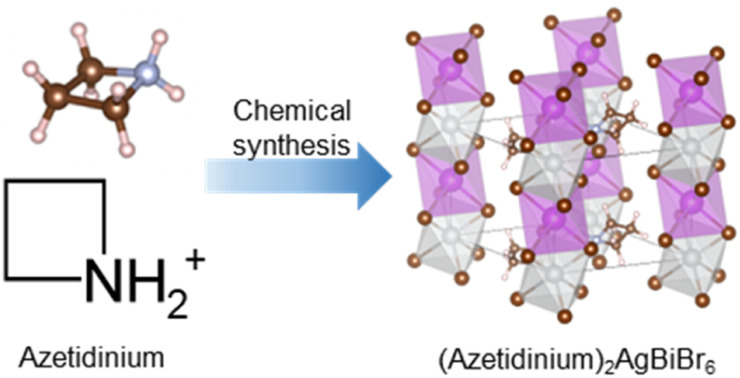

## Introduction

Organic–inorganic hybrid halide perovskites show excellent photovoltaic properties in solar cells.^1–4^ The power conversion efficiency (PCE) of photovoltaic cells using halide perovskite layers has reached 26.1% now.^5^ At the dawn of the perovskite solar cell research, CH_3_NH_3_PbI_3_ (methylammonium lead iodide, MAPI) and CH(NH_2_)_2_PbI_3_ (formamidinium lead iodide, FAPI) have been widely employed. However, a number of fundamental problems must still be solved for successful commercialization, in particular the instability due to exposure to light, moisture, and oxygen.^6–9^ Moreover, conventionally used lead halide perovskites pose a threat to human health and the environment because of the toxicity of lead.^10^ As perovskite materials are versatile, the above-mentioned hurdles may be solved by material composition design using new halide perovskite compositions.^11–13^

The chemical formula of fundamental hybrid organic–inorganic perovskite is ABX_3_, where A is an organic or inorganic monovalent cation such as Rb, Cs, methylammonium (MA), or formamidinium (FA), B is a divalent metal ion and X is a halide, such as Cl, Br, or I. The halide perovskites based on MA and FA are the best-known and best performance light absorbers. Their exploitation now mostly concerns device engineering.^2,14,15^ Triple-cation based lead halide perovskites were suggested by Saliba *et al.*^16^ to resolve the stability issue leading to both better stability and higher PCE compared to solar cells with only a single organic cation, MA or FA. The A-site cation plays a critical role in determining the position of the B–X octahedra, dramatically influencing the bandgap value.^17^

Lead substitution has also been considered a significant matter due to the toxicity of this element. Alternatively, the divalent ions from group IV, Sn^2+^ and Ge^2+^ could be a good option to build an environmentally friendly halide perovskite.^18,19^ However, it has been challenging to obtain a homogeneous thin film of Sn^2+^-based perovskite, and the PCE of the devices is still poor as well as their stability.^18,20,40^ In the case of Ge^2+^-based perovskites, they tend to have an indirect and wide bandgap which leads to inefficient light absorption.^7,21^

We have recently focused on the halide double perovskite structure (A_2_B′B′′X_6_) where B′ is a monovalent metal ion (B′^+^) and B′′ is a trivalent metal ion (B′′^3+^) in order to avoid toxic lead.^22–24^ Among the halide double perovskites with Ag^+^ and Bi^3+^ as B′ and B′′, Cs_2_AgBiBr_6_ and Cs_2_AgBiCl_6_ were the first compositions to be synthesized.^25,26^ Cs_2_AgBiBr_6_ has a cubic structure and decent light absorption, as well as high photoconductivity and a relatively suitable bandgap which makes it a good candidate to be used in photovoltaic cells.^23,27^ According to the Goldschmidt tolerance factor approach for the prediction of the perovskite structure, which is given as *t* = (*r*_A_ + *r*_X_)/(2^1/2^[*r*_B_ + *r*_X_]), the value of average *t* for Cs_2_AgBiBr_6_ approximates 1 which predicts a cubic lattice structure, while a local lattice distortion can appear due to the disposition of adjacent Ag^+^ and Bi^3+^.^26,28^ Cs_2_AgBiBr_6_ has an indirect bandgap of 1.95 eV.^25,27^ However, the large values of the effective masses of the charge carriers may be a reason for the limited PCE of 10% for a photovoltaic device. We have also explained this limitation in the context of the much larger screening effects in the hybrid organic counterparts.^29^ Also, no substantial increase of stability is expected.^30^ Cs_2_AgBiI_6_ is rarely studied because its synthesis is difficult, and therefore there has been no report on detailed structural characterization of it, but it can be assumed that it has a higher likelihood to have a direct bandgap than Cs_2_AgBiBr_6_ or Cs_2_AgBiCl_6_.^30–32^ Still the photovoltaic cells having hybrid lead halide perovskite as an absorbing layer have significantly larger PCE than the ones with Cs-based all-inorganic double halide perovskites.^17,23^ Finding appropriate organic A-site cations seems to be rather critical to design suitable compositions with ordered structure. Several research groups have started to investigate hybrid organic–inorganic halide double perovskite systems considering organic A-site cations. Nanocrystal synthesis of (MA)_2_AgBiBr_6_ and (MA)_2_AgBiI_6_ has been performed, but the fabrication of thin films and its application in solar cells are still in progress and experimentally unclear due to the formation of the preferential phase (MA)_3_Bi_2_X_9_.^33–35^ Synthesis of (FA)_2_AgBiBr_6_ and (FA)_2_AgBiI_6_ was attempted by Wei *et al.*, but the reactions also lead to a preference for the formation of each (FA)_3_Bi_2_X_9_ phase separately.^33^

We adopted the (CH_2_)_3_NH_2_^+^ ion (azetidinium, Az) as one of the candidate molecules, which can become a feasible option, because its effective radius is computationally calculated as 250 pm, which is insignificantly smaller than FA (253 pm) but larger than MA (217 pm).^11^ Pering *et al.* have initially shown that (CH_2_)_3_NH_2_PbI_3_ (azetidinium lead iodide, AzPbI_3_) thin film and its mixture with MAPI can be employed as a light absorber with good stability, showing a PCE of 1.15% in a photovoltaic cell.^36^ Nanocrystals of AzPbI_3_ and AzPbBr_3_ have been synthesized by a few groups and characterized by structural and chemical analysis suggesting that it could be a promising light absorption layer.^37,38^ Nevertheless, at present the experimental research on this cation is insignificant. Even though AzPbI_3_ was proven to be a light-absorbing semiconductor with optical bandgap of 2.15 eV,^36^ its low absorbance is a limiting factor for the use of this material in solar cells. Furthermore, little is known about thin film deposition to fabricate high quality photovoltaic devices. The Az-ion has rarely been studied as a component of the perovskite phase. On the other side, the pioneering thin film of AzPbI_3_ has proven to have better moisture stability compared to both MAPI and FAPI, and it seems to hold true for other Az lead perovskite systems including AzPbBr_3_ and AzPbBr_*x*_I_3−*x*_.^36–39^ Since the improvement of stability is a very important issue, the compositional design with the Az ion was considered worth pursuing to obtain a new stable light harvesting material. By substituting the Az-cation into Cs_2_AgBiBr_6_, we propose the possibility of broadening the use of halides as light absorbers and introduce the azetidinium Ag–Bi double metal ion system (Az)_2_AgBiX_6_. Herein, we explore (Az)_2_AgBiBr_6_ as a first easily synthesized variant. The synthesis of crystalline powders, and the deposition of textured thin films on glass were performed and characterized using X-ray diffraction and UV-vis spectrophotometry. (Az)_2_AgBiCl_6_ and (Az)_2_AgBiI_6_ have also been considered to be promising candidates to explore, however, in the case of hypothetic (Az)_2_AgBiI_6_, the synthesis led to a segregation of Ag and Bi forming (Az)_3_Bi_2_I_9_ and a second phase containing Ag and I. This system is still under investigation. (Az)_2_AgBiCl_6_ has to be preferentially solved, a problematic status that has shown poor solubility of its precursor in polar solvents such as *N*,*N*-dimethylformamide (DMF) and dimethyl sulfoxide (DMSO). So we have restricted the present work to (Az)_2_AgBiBr_6_ for which the synthesis is clearly feasible and data well determined.

## Results and discussion

### Synthesis

We synthesized polycrystalline powders, because single crystal formation of (Az)_2_AgBiBr_6_ turned out to be difficult. The powder of (Az)_2_AgBiBr_6_ used for diffraction experiments was obtained by an evaporation method and has a bright yellow color. Bismuth(iii) bromide (BiBr_3_) (97%) was purchased from Sigma-Aldrich, silver bromide (AgBr) (99.5%) from Alfa Aesar, and hydrobromic acid (HBr) (48%) from Sigma-Aldrich. All chemicals were used without further purification. The powder of AzBr was synthesized by HBr and azetidine (98%, purchased from Alfa Aesar). 11 g of HBr were added to 5 g of azetidine on an ice bath (approx. 0 °C). The obtained powders and liquid solution were dried in a rotary evaporator until leaving a bright yellow powder. The powder was washed using diethyl ether to remove excess of bromine and then recrystallized using isopropanol. ^1^H NMR spectra of the obtained product are shown in Fig. S1.†

The polycrystalline powders of (Az)_2_AgBiBr_6_ were produced by the solvent evaporation method. First, AzBr, BiBr_3_ and AgBr were dissolved in HBr *via* magnetic stirring at 60 °C at 400 rpm. The precursor solution was filtered with a PTFE (polytetrafluorethylene) membrane filter of 0.7 μm pore size and poured into Petri dishes, which were covered with glass covers to enable slow evaporation. Then the solution was heated to 100 °C and left on the hot plate for two to three days at a constant temperature of 100 °C until the crystalline powder was formed. In the last step, it was ground to obtain a homogeneous powder.

The thin films were prepared by the sol–gel method. The precursor solution was acquired by stirring 1 mmol of AzBr, 0.5 mmol of AgBr, and 0.5 mmol of BiBr_3_ in 0.5 ml of DMSO for 90 minutes. The dissolved precursor was filtered with a PTFE membrane filter of 0.45 μm pore size. The glass substrate was cleaned *via* ultra-sonicating in acetone for 15 minutes, distilled water for 15 minutes, and ethanol for 15 minutes. The cleaned glass was exposed to a UV–ozone treatment for 30 minutes for better diffusion of the precursors. The (Az)_2_AgBiBr_6_ thin film was deposited from the filtered precursor *via* one step spin-coating at 2000 rpm during 30 s onto the glass substrate. Then it was annealed at 125 °C for 30 minutes on a hot plate in a N_2_-filled glove box.

### Crystal structure

**Fig. 1:**
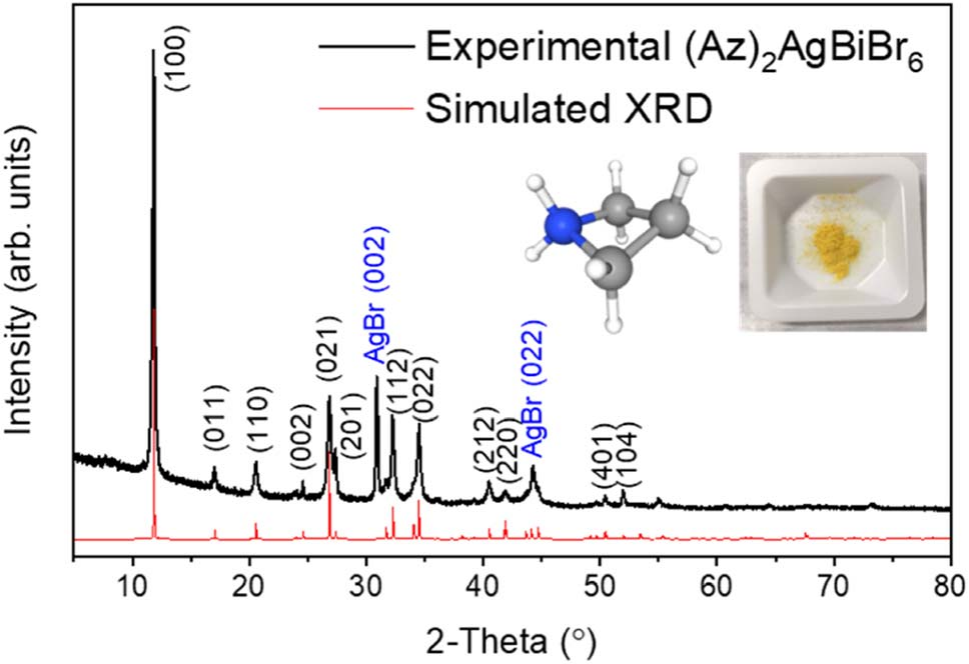
XRD patterns from experiment (black line) and Rietveld refinement (red line) with pictures of azetidinium [(CH_2_)_3_NH_2_]^+^ molecular cation and (Az)_2_AgBiBr_6_ polycrystalline powder.

The crystal structure of (Az)_2_AgBiBr_6_ was determined by X-ray powder diffraction (Fig. 1). For a successful refinement of the initial structure model, it was necessary to use anisotropic displacement parameters for the heaviest atom (Bi). Isotropic displacement parameters were assigned to all other atoms.

**Fig. 2:**
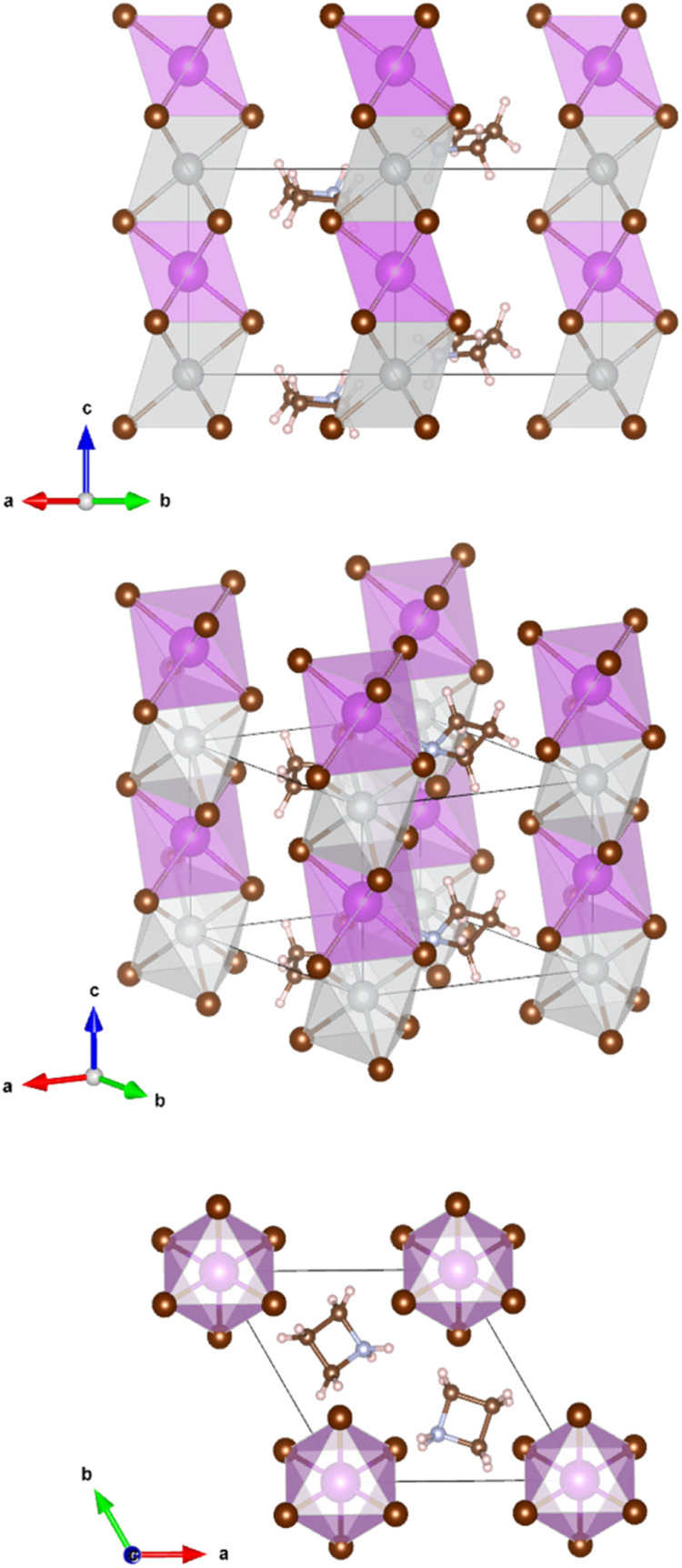
Refined crystal structure of (Az)_2_AgBiBr_6_, crystal symmetry *P*3̄*m*1. The disordered heterocyclic molecules used to simulate the azetidinium cation are arranged in a specific occupied position. (3 carbons – small brown spheres, one nitrogen – small blue spheres, 8 hydrogens – small bright red spheres) big brown spheres indicate bromide ions, grey spheres – Ag atoms, and purple spheres – Bi atom.

The crystals are trigonal with space group *P*3̄*m*1and contain a one-dimensional (1D) structure as shown in Fig. 2. The inorganic part of the structure consists of rods of interconnected [AgBr_6_] and [BiBr_6_] octahedra which run parallel to the *c*-axis. Each octahedron shares common faces with two neighboring octahedra in a rod. The [AgBr_6_] and [BiBr_6_] octahedra alternate but considerable disorder concerning a strict Ag–Bi–Ag–Bi sequence is observed. According to the structure refinement, the B1 site is occupied by 59% Ag and 41% Bi while the B2 site is occupied by 41% Ag and 59% Bi. It can be assumed that some rods are shifted by 0.5*c*_0_ relative to others along the *c* axis. Although Ag^+^ and Bi^3+^ possess similar ionic radii (1.17 Å and 1.03 Å, respectively), it is unlikely that Ag^+^ and Bi^3+^ partly replace each other in a given rod due to the high charge of the Bi^3+^ cations making a direct neighborhood among them very unlikely. The octahedra are slightly distorted with Br–B1(Ag dominated)–Br angles of 82.3° and 97.7° and Br–B2(Bi dominated)–Br angles of 87.8° and 92.2°. This means, the octahedra are elongated along the *c* axis. The bond lengths are *d*(B1–Br) = 2.97 Å and *d*(B2–Br) = 2.82 Å.

The Az^+^ cations take specific sites in the crystal lattice intercalated between the rods of octahedra; the individual Az^+^ cations are, however, disordered. They probably rotate at room temperature about their center points. It was, therefore, impossible to distinguish between the carbon and nitrogen atoms of the ring. Hydrogen atoms could not be located.

AgBr was detected as a secondary phase which might have been left in the sample caused by the imperfect solubility of its powder. Depending on different solvents and growth temperatures, it has shown either complex phases or a AgBr residue. This question is still open to further research. The solid solution method was also attempted by mixing each reactant powder, however, it was difficult to obtain a high purity compound due to the appearance of several phases and a large amount of AgBr residue.

### Thin films

**Fig. 3:**
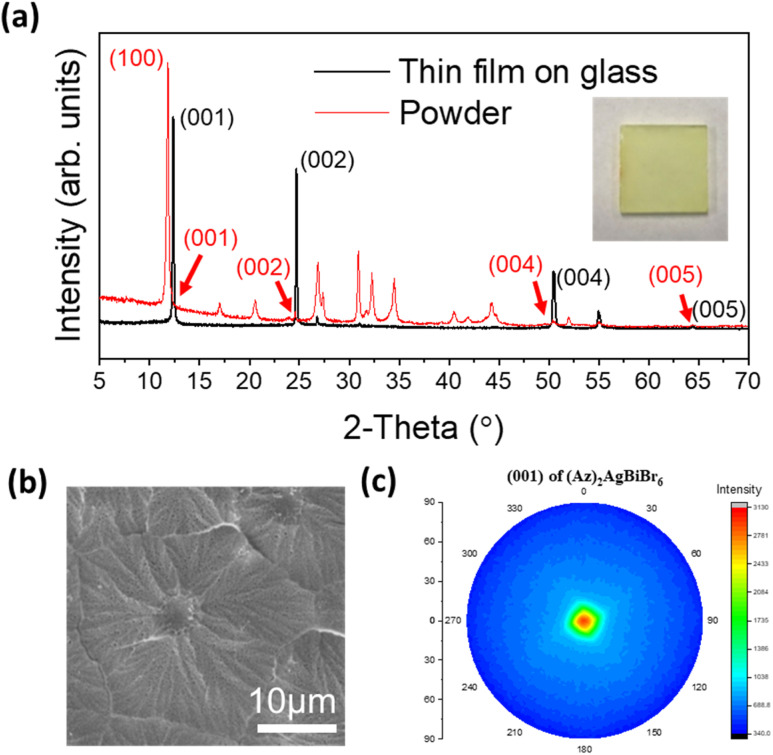
(a) XRD patterns and pictures of optimized (Az)_2_AgBiBr_6_ thin film on glass, (b) morphology characteristics through top-view SEM image, and (c) pole figure diagram of (001) reflection of (Az)_2_AgBiBr_6_ thin film, which is a predominant reflection in its pattern.

Thin film growth on purified glass was done *via* spin-coating in a N_2_ filled glove box. In the case of (Az)_2_AgBiBr_6_, the diffraction pattern of the film presents predominantly the (00*l*) reflections at 12.36°, 24.69°, 50.43° and 64.30° (weak) corresponding to the (001), (002), (004) and (005) reflections, respectively. All other peaks are very weak. This is consistent with a parallel growth of the thin film with the *c*-axis of all crystals being nearly perfectly oriented perpendicular to the glass plane. In contrast, the (100) reflection is predominant in the XRD pattern of the powder which was recorded from a sample with statistically arranged crystals (Fig. 3a). According to the lattice diagram presented above, the preferred growth in the thin film should correspond to the orientation of stacking of [AgBr_6_] and [BiBr_6_] octahedra.

Additional weak intensities around 26.7°, 51.8° and 54.9° can either be assigned to the (201), (104) and (114) reflections of (Az)_2_AgBiBr_6_, in accordance with the XRD pattern of the (Az)_2_AgBiBr_6_ powder or might represent the (111), (311) and (222) reflections of AgBr residues. Even though the thin film was optimized, in some samples a tiny additional peak appeared on the left side of the strong (001) peak which is indexed as the (100) reflection of (Az)_2_AgBiBr_6_ (Fig. S3, left†) indicating a slightly reduced preferred orientation of the crystals. The (100) reflection was somewhat prominent in incomplete thin films. The rotational speed of the spin coating process higher than 2000 rpm and the lower temperature than 125 °C tend to influence the appearance of the (100) reflection. This shows incomplete growth on the glass substrate (Fig. S3, right†). We identified that the compositional ratio of N : Ag : Bi : Br presents 2 : 1 : 1 : 8 through EDX. This is not the exact expected 2 : 1 : 1 : 6 ratio (Fig. S4†). The columnar growth of the films appears to drive some of excess Br onto the surface where the electron microscope is highly sensitive. With the morphology observation, the optimized thin film is macroscopically opaque and completely compact on the microscale. The grain of the layer seems to be close to flower-like shape on SEM images (Fig. 3b). One densified grain has a mean diameter of several micrometers until the formation of grain boundaries. We additionally confirmed that grains may have inhomogeneous Bi distribution through linear EDX spectra (Fig. S5†).

The four (00*l*) peaks in the XRD pattern of the thin film are sharp indicating high crystallinity. This was not expected for epitaxial growth when the layers were deposited on glass. The pole figure diagram was collected using the (001) reflection in the thin film XRD pattern (Fig. 3c). The pole figure with a single spot of high intensity in the center again confirms that the layer is highly textured on glass, and it may indicate that the film can potentially be grown on a flat surface of various amorphous substrates.

### Optical properties

**Fig. 4:**
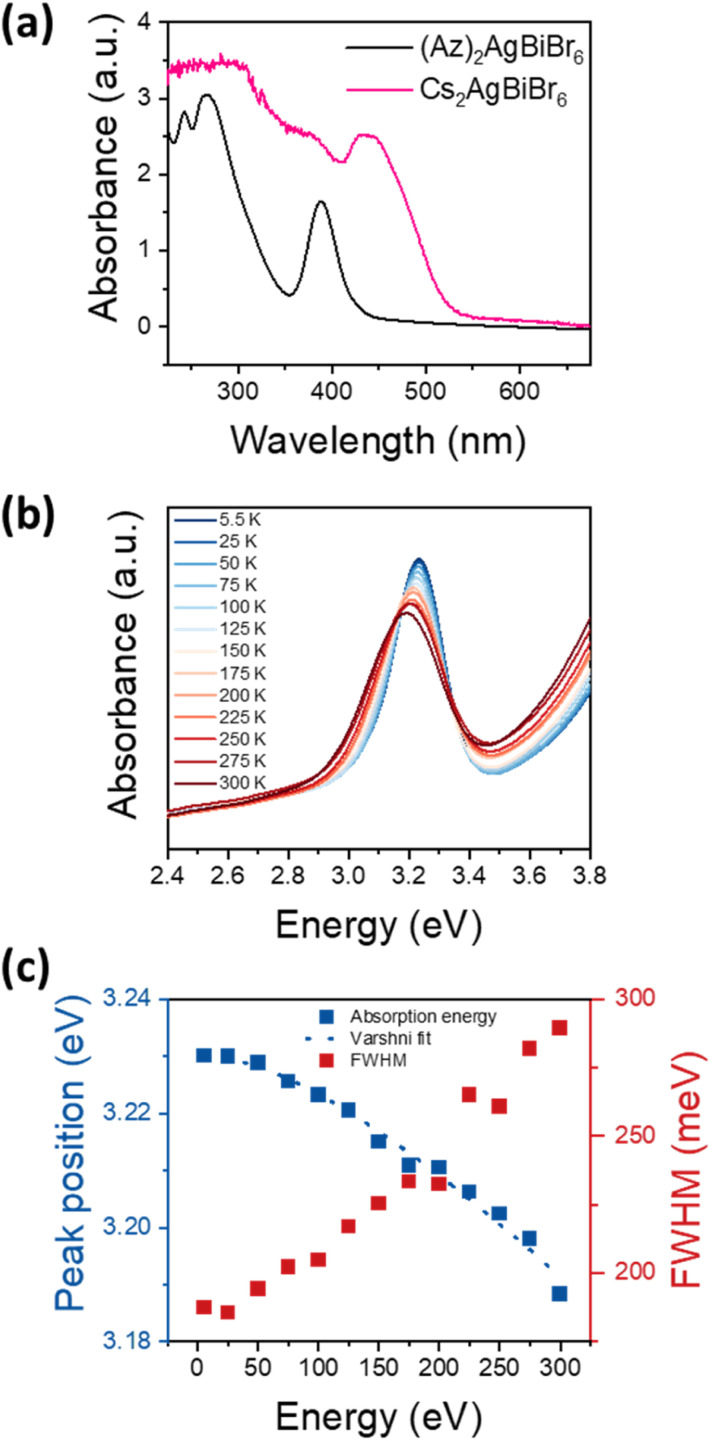
(a) Absorbance spectra of (Az)_2_AgBiBr_6_ thin film in comparison with analogous double perovskite Cs_2_AgBiBr_6_, (b) temperature dependent absorbance spectra of (Az)_2_AgBiBr_6_. (c) Peak position and FWHM of the excitonic peak at temperatures between 5.5 K and 300 K. The peak position is fit according to *E* = *E*_0_ + (*αT*^2^)/(*β* + *T*) with *E*_0_ = 3.230, *α* = 2.34 × 10^−4^ eV K^−1^ and *β* = 246 K.

Optical bandgap approximation was assessed by the absorbance of thin films as shown in Fig. 4a, in comparison to the absorbance of a Cs_2_AgBiBr_6_ thin film.

The absorbance spectrum of (Az)_2_AgBiBr_6_ depicts a dominant peak at 389 nm and two additional local maxima below 300 nm. The dominant absorption peak at 389 nm or 3.188 eV, respectively, can be described with a Gaussian function with a full-width-at-half-maximum (FWHM) of 301 meV (Fig. S6†). The shape and distinctiveness of the peak indicate that it may be of excitonic origin. Based on our conclusions about the structure of the (Az)_2_AgBiBr_6_, we expect the charge carriers to exhibit an 1D confinement within the rod-like pillars of inorganic [AgBr_6_] and [BiBr_6_] octahedra and thus the appearance of excitonic peaks in the absorbance spectrum is likely. However, a similar absorption peak occurring in Cs_2_AgBiBr_6_ has recently been identified as non-excitonic, as it neither shifts with temperature nor with carrier confinement in quantum dots.^41–43^ Instead, the absorption peak in Cs_2_AgBiBr_6_ is attributed to either an internal Bi–Bi transition or to a Ag–Bi charge transfer transition.^41^ Note that Cs_2_AgBiBr_6_ crystallizes in a 3D double-perovskite structure consisting of the same inorganic component as our low-dimensional perovskite-like material.

To clarify whether the peak observed at around 3.2 eV is excitonic, we performed temperature-dependent absorbance measurements shown in Fig. 4b for temperatures between 5.5 K and 300 K. With increasing temperature, the absorption peak at 3.2 eV broadens and shifts towards lower energies, indicating that the transition originates from electronic bands rather than from elemental orbitals. This confirms that the peak is indeed an excitonic transition. Fig. 4c depicts the energetic positions and FWHM of the main peak between 5.5 K and 300 K with a total shift of 42 meV. As common for the bandgap of a semiconductor, the temperature-dependent shift can be well described with the Varshni formula. Assuming a layer thickness between 100 nm and 1 μm, the absorption coefficient can be estimated as 10^4^ cm^−1^ to 10^5^ cm^−1^, which indicates a direct semiconductor. Note that Cs_2_AgBiBr_6_ is considered to exhibit an indirect bandgap.^23,24^ However, Connor *et al.* previously reported that dimensional reduction of the [AgBr_6_] and [BiBr_6_] octahedra network in Cs_2_AgBiBr_6_ and its structural distortion drive indirect-to-direct bandgap transition by dimensional confinement.^44^ (Az)_2_AgBiBr_6_ adopts a 1D network, therefore it can be immensely influenced by the effects through dimensional reduction. Accordingly, we assume the absorption peak at 3.2 eV to be a direct bandgap excitonic transition.

### DFT simulations

In addition to measuring the various physical properties of (Az)_2_AgBiBr_6_, we have also performed the corresponding first-principles calculations based on density functional theory (DFT). The calculations were carried out within the projector augmented-wave (PAW) formalism using the CP-PAW program.^45,46^ The exchange–correlational energy functional is approximated within the generalized-gradient approximation (GGA) framework in the form of the well-known Perdew–Burke–Ernzerhof (PBE) functional.^47^ For sufficiently accurate calculations, we choose a plane wave cut-off of 60 Ry for the wave functions and 240 Ry for the charge density. For the generation of the *k*-point mesh, we choose the plane wave cut-off for the Fourier interpolation to be *R* = 40 in the physical length scale, with the distance between successive *k*-points being 2π/*R*. The *k*-point grid includes the *Γ*-point, which is the standard choice. The Brillouin-zone integration was performed with the linear-tetrahedron method, with the Blöchl corrections.^48–50^

As mentioned in the section “Crystal structure”, the space group of (Az)_2_AgBiBr_6_ is *P*3̄*m*1, which consists of the hexagonal unit cell. The coordination number of Ag and Bi atoms is 6, forming an octahedral structure with 6 nearest Br neighbours. We used the obtained experimental lattice parameters taken from Table 1 and performed our calculation within the single unit cell. The exact coordinates of all the 32 atoms embedded in the unit cell are provided in the “.strc” section (along with a pictorial representation) of the ESI note (Fig. S7).† The reader should note that we choose the orientation of the Az^+^ cations such that the nitrogen ion, suspected of being electron-deficient, is the closest to the electron-rich bromide neighbours.

**Table 1:** Experimental and crystallographic parameters for the structure refinements of (Az)_2_AgBiBr_6_

Diffractometer	STOE StadiMP with Mythen 1K detector
Wavelength	1.54059 Å
Sample holder	0.3 mm glass capillary
2*θ* range of data used [°]	5.0–90.00
Step size [°2*θ*]	0.0150
No. contributing reflections	163
No. geometric restraints	3
No. structural parameters	13
No. profile parameters	14
FWHM at *ca.* 24°2*θ* [°2*θ*]	0.07–0.34
*R*_I_	0.048
*R*_F_	0.045
*R*_wp_	0.123
*χ* ^2^	2.35
Space group	*P*3̄*m*1 (no. 164)
*a* [Å]	8.6190 (1)
*b* [Å]	8.6190 (1)
*c* [Å]	7.2416 (1)
*V*_UC_ [Å^3^]	465.89 (1)
Density (calc.) [g cm^−3^]	3.25
Unit cell content	(Az)_2_AgBiBr_6_

A set of projector functions for each atom is given for the augmentation. For our simulation, we have used (i) two projector functions per angular momentum up to *l* = 2 for the bromide, silver, nitrogen and carbon species, (ii) two projector functions per angular momentum up to *l* = 3 for the bismuth species (to account for the presence of the f-states), and (iii) two projector functions per angular momentum up to *l* = 1 for the hydrogen species. We allow all the atoms in our simulated (Az)_2_AgBiBr_6_ to be electronically relaxed but only allow the Az^+^ cations to be rotated about their center point in the subsequent atomic relaxation step. Within this limited atomic relaxation, the total energy converges to a particular value, after which no significant deviation occurs when we remove all friction parameters to the atom and electrons.

**Fig. 5:**
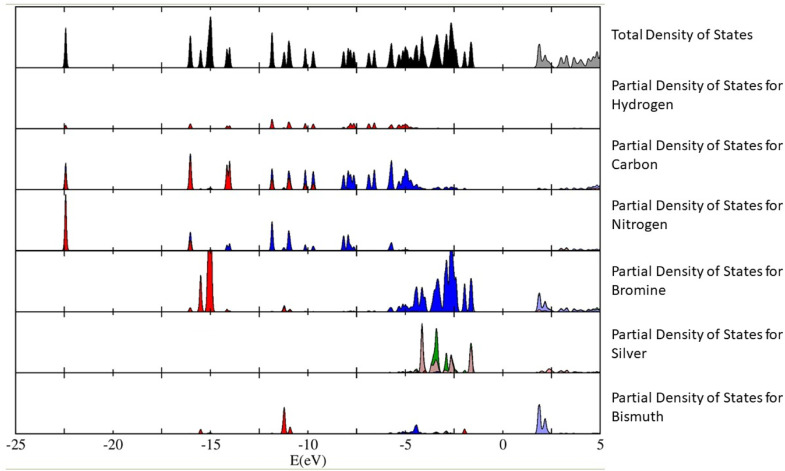
The total density of states of (Az)_2_AgBiBr_6_ is plotted on the top graph (black-filled and gray-empty), with the partial density of states for each species of hydrogen, carbon, nitrogen, bromine, silver and bismuth plotted in succession. In the *x*-axis, the Fermi level is chosen as the zero of the energy axis, which could also be called the “mid-gap energy”. In the partial density of states, we have denoted in colour, the nature of each orbital contributing to each value of energy in the *x*-axis. They are: red – s orbitals, blue – p orbitals, dark green – d-e_g_ orbitals, brown – d-t_2g_ orbitals and orange: f orbitals. The *y*-axis is taken to be in arbitrary units but note that the scale of the partial density of states for each species is 1/3 of the total density of states.

The calculations have revealed that the nature of the bandgap of (Az)_2_AgBiBr_6_ (with the above-mentioned orientation of the Az^+^ cations) is direct, with a value of 3.0550 eV. This value is slightly lower than the empirical bandgap of thin film at 5.5 K, which is roughly 3.08 eV. We have plotted out the density of states (and the partial density of states contributing to the total density of states) of the (Az)_2_AgBiBr_6_ system (Fig. 5). We can infer from Fig. 5 that the p-orbitals of the bromine and the d-orbitals of silver predominantly contribute to the valence band of (Az)_2_AgBiBr_6_, while t_2g_ d-orbitals and the p-orbitals of the bismuth and bromine contribute to the conduction band.

According to the plot of the partial density of states, we assume that there can be a sub-bandgap state since the ones of bromine and silver are closely calculated to the value of the total density of states. It can be conflicting with the estimation of an excitonic state existence as mentioned above.

### Stability

Stability tests were performed to examine the accessibility for optoelectronic or photovoltaic device. Only thin films on glass were tested. The samples have been located immediately next to a window, where the sunlight exposure has been coming from the south (location: 51° 28′ 32.664′′ N, 7° 2′ 17.16′′ E, Germany) during spring season. It is presumed that the exposure was for 8 hours with strong light incidence for 3 hours in the middle of the day apart from several rainy days, while the dark condition has been maintained during the remaining hours of each day periodically. The record of humidity and temperature was performed daily.

The resulting (Az)_2_AgBiBr_6_ has no evident change in absorbance spectra until 57 days of exposure to the ambient while it became darker when examined with naked eye (Fig. S8†). According to the XRD pattern comparison, no changes in the XRD diffractogram is observed within 62 days. Within this timeframe there is no distinctive chemical reaction. In addition, there is an increase of absorbance at the intermediate peak between pristine and after 9 days. This can be probably originated by the formation of intermediate structures. These changes cannot be detected by XRD due to its very low concentration. We also did not see any change in the color of the film at this time.

For comparison in detail, we measured the XRD pattern of the same film which was kept in ambient conditions during 16 months after the stability test. The pattern (Fig. S8c†) presents an increase in AgBr peak intensity, which means that the material is decomposing. Also unknown reflections are detected that do not correspond to any reactants, however their intensities are too low for further specific structure determination.

Thermal stability has been tested on a hot plate in an N_2_ filled glove box. The tests were performed with several samples heated for 5 minutes at specified temperatures, ranging from 130 °C to 200 °C with 10 °C intervals. XRD measurements for the samples were performed at room temperature right after heating. The result shows that the thin film retained a stable state, but peak segregation of (100) occurred at 150 °C. The emergence of reflections estimated as (Az)_3_Bi_2_Br_9_ at 8.36° with its preferred orientation at 16.81° and 25.42° starting from 160 °C. They coexist in the range between 160 °C and 190 °C. All reflections mostly vanish at 200 °C as a degradation. No reflections were observed for AzBr or BiBr_3_ between 160 °C and 190 °C, and AgBr reflections tend to be the result of decomposition.

## Conclusions

It was demonstrated that azetidinium (Az^+^), a heterocyclic ammonium cation can be an alternative as an organic A-site of an organic–inorganic hybrid bromide double perovskite system. (Az)_2_AgBiBr_6_ adopts a 1D system with high disorder of Az^+^. The thin film on glass appears to have highly oriented crystallinity with only a few predominant reflections. In our experimental dataset, the most remarkable aspect is that this material indicates a direct bandgap semiconductor with an excitonic state at around 3.2 eV. Meanwhile, our DFT simulation can imply that the intermediate band is even a sub-bandgap state. This should be revealed with further research. The successful synthesis of (Az)_2_AgBiBr_6_ can be considered as an important factor to widely exploring a reasonable novel organic–inorganic hybrid halide double perovskite system. This route also makes an extension to explore mixed halide for a possibility of bandgap tunability, such as (Az)_2_AgBiBr_6−*x*_I_*x*_ or (Az)_2_AgBiBr_6−*x*_Cl_*x*_. It may not be applicable to better photovoltaic performance, but it can be applied to a photodetector field due to its peculiar optical property. Moreover, it may become an essential opportunity helping to progress further research for optoelectronics and photovoltaics.

## Author contributions

Y. U. J. developed this line of research and synthesized the powders as well as the films. B. M. and A. N. S. did the structure refinement, B. M. finally resolved it. I. C. J. Y. performed DFT simulations. A. D. helped with optical analysis. K. W. helped with morphological analysis. A. D. K. and M. E. C. helped with synthesis, analytic characterization, and revision. L. S. and F. M. performed temperature dependent UV-vis absorbance measurement and helped with its analysis. N. B. and D. C. L. helped with data interpretation and text.

## Conflicts of interest

There are no conflicts to declare.

## Supplementary Material

RA-013-D3RA05966A-s001

RA-013-D3RA05966A-s002
