# Supplementary material for: Lead-free organic–inorganic azetidinium alternating metal cation bromide: [(CH2)3NH2]2AgBiBr6, a perovskite-related absorber
Source: RSC Adv. 2023 Dec 12;13(51):36079–87. doi: 10.1039/d3ra05966a (PMC10714204; doi:10.1039/d3ra05966a)
Supplement: RA-013-D3RA05966A-s001 [file RA-013-D3RA05966A-s001.pdf]

Electronic Supplementary Material (ESI) for RSC Advances.
This journal is © The Royal Society of Chemistry 2023

Lead-free organic-inorganic azetidinium alternating metal cation bromide: $[(CH_2)_3NH_2]_2AgBiBr_6$, a perovskite-related absorber

Young Un Jin,[a] Bernd Marler,[b] Andrei D. Karabanov,[a] Kristina Winkler,[c,d] Ian Chang Jie Yap,[a] Astita Dubey,[a] Leon Spee,[e,f] Marianela Escobar Castillo,[a] Franziska Muckel,[e] Andrei N. Salak,[g] Niels J. Benson,[c] and Doru C. Lupascu*[a]

a. Institute for Materials Science and Center for Nanointegration Duisburg-Essen (CENIDE), University of Duisburg-Essen, 45141 Essen, Germany

b. Institute of Geology, Mineralogy and Geophysics, Ruhr-University Bochum, 44780 Bochum, Germany

c. Germany Institute of Technology for Nanostructures (NST), University of Duisburg-Essen, 47057 Duisburg, Germany

d. Fraunhofer Institute for Solar Energy Systems (ISE), 79110 Freiburg

e. Electroenergetic Functional Materials (EEFM) & CENIDE, University Duisburg-Essen, 47057 Duisburg, Germany

f. Werkstoffe der Elektrotechnik (WET) & CENIDE, University Duisburg-Essen, 47057 Duisburg, Germany

g. Department of Materials and Ceramics Engineering, CICECO-Aveiro Institute of Materials, University of Aveiro, 3810-193 Aveiro, Portugal.

Material preparation

AzBr was synthesized, bismuth (III) bromide ($BiBr_3$) (97%) was purchased from Sigma-Aldrich. Silver bromide (AgBr) (99.5%) was purchased from Alfa Aesar. Hydrobromic acid (HBr) (48%) was acquired from Sigma-Aldrich. All chemicals were used without further purification.

Methods

AzBr: The powder of AzBr was synthesized by HBr and Azetidine (98%, purchased from Alfa Aesar). 11g of HBr was added to 5g of Azetidine on the ice bath where it is nearby 0°C. The obtained powders and liquid solution were dried by rotary evaporator until leaving bright yellow powder. It was washed with diethyl ether to remove excess of bromine and then recrystallized using isopropanol. ^{1}H NMR spectra of the obtained product is shown in Fig. S1.

$(Az)_2AgBiBr_6$: The powders were produced by the solvent evaporation method. First, AzBr, $BiBr_3$ and AgBr were dissolved in HBr via magnetic stirring at 60°C at 400 rpm (rounds per minute). The precursor solution was filtered with a PTFE (polytetrafluorethylene) membrane filter of 0.7 µm pore size and poured into Petri dishes, which were covered with glass covers to enable slow evaporation. Then the solution was heated to 100°C and left on the hot plate for 2-3 days at constant temperature of 100°C till the crystalline powder was formed. In the last step it was grinded to get a homogeneous powder.

The thin film was obtained by sol-gel method. The precursor solution was acquired by 1mmol of AzBr, 0.5mmol of AgBr, and 0.5mmol of $BiBr_3$ in 0.5ml of DMSO stirring for 90 minutes. Dissolved precursor was filtered with a PTFE membrane filter of 0.45 µm pore size. The glass substrate was prepared with cleaning procedure through the ultra-sonicating in acetone for 15 minutes, distilled water for 15 minutes, and ethanol for 15 minutes. The cleaned glass was exposed to UV-ozone treatment equipment for 30 minutes for better diffusion of the precursors. $(Az)_2AgBiBr_6$ thin film on glass substrate was deposited from the filtered precursor via spin-coating process with 2000rpm during 30s as 1-step, and then it was annealed at 125°C for 30 minutes on the hot plate in a N_2 filled glove box.

X-ray Diffraction and Pole figure measurement

Diffraction data for the structure analysis were collected on a STOE StadiMP powder diffractometer in modified Debye-Scherrer geometry using Cu Kα1 radiation (λ = 1.54059 Å). The sample was sealed in a borosilicate glass capillary with a diameter of 0.3 mm to avoid preferred orientation of the crystals. The structural model was refined using the FullProf 2K program [xx] with scattering factors as implemented there. For the Rietveld refinement, three soft distance restraints were used on the carbon atoms to refine the azetidinium cation as a pseudo rigid body, d(C-C) = 1.54(1) Å. Six additional parameters were necessary to describe the anisotropic peak widths.

X-ray diffraction measurement of the thin film was performed using the PANalytical X'pert PRO diffractometer (Cu K-α1 radiation, 1.540598 Å) equipped with PIXcel3D-Medipix3 detector using a scanning line detecting mode (0.04 rad soller slit, 1/8° fixed divergence slit, 10.8 mm mask, 9.1 mm anti-scatter slit were used on the incident beam path, and 0.04 rad soller slit was used on the diffracted beam path). Pole figure measurement was performed using Euler chi-phi xyz stage 240mm for each reflection peaks of thin films fixed onto center of the stage. Continuous scanning mode was used for every orientation of which the polar angle χ (chi) was ranged from 0° to 90° with 1° step size, and the azimuthal angle ϕ (phi) was scanned ranging from 0° to 360° for 1 second per 1° step. XRD analysis and crystal structure determination were performed using Highscore plus software produced by PANalytical, and pole figure analysis was performed using X'pert Texture software.

Scanning Electron Microscopy (with EDX)

Scanning electron microscopy experiment was performed by the equipment from Jeol (JSM-7500F). The samples were gold-coated by vacuum vapour deposition prior to analysis.

UV-Vis Spectroscopy

Absorbance spectra were acquired from thin film samples on glass using a Shimadzu UV2600 UV-vis spectrophotometer (Japan). For temperature-dependent measurements, the sample is placed in a Janis ST-500-SNOUT cryostat. For setting the temperature a LakeShore Model 335 temperature controller was used. After reaching the lowest temperature, 30 minutes were waited and afterwards the measurements were made from low to high temperatures. $BaSO_4$ powder was used for the reference of reflection data. For bandgap estimation Tauc plot was directly extracted by % diffuse reflectance spectra of powder samples and absorption spectra of thin films through the conversion to direct transition to clarify its bandgap nature.

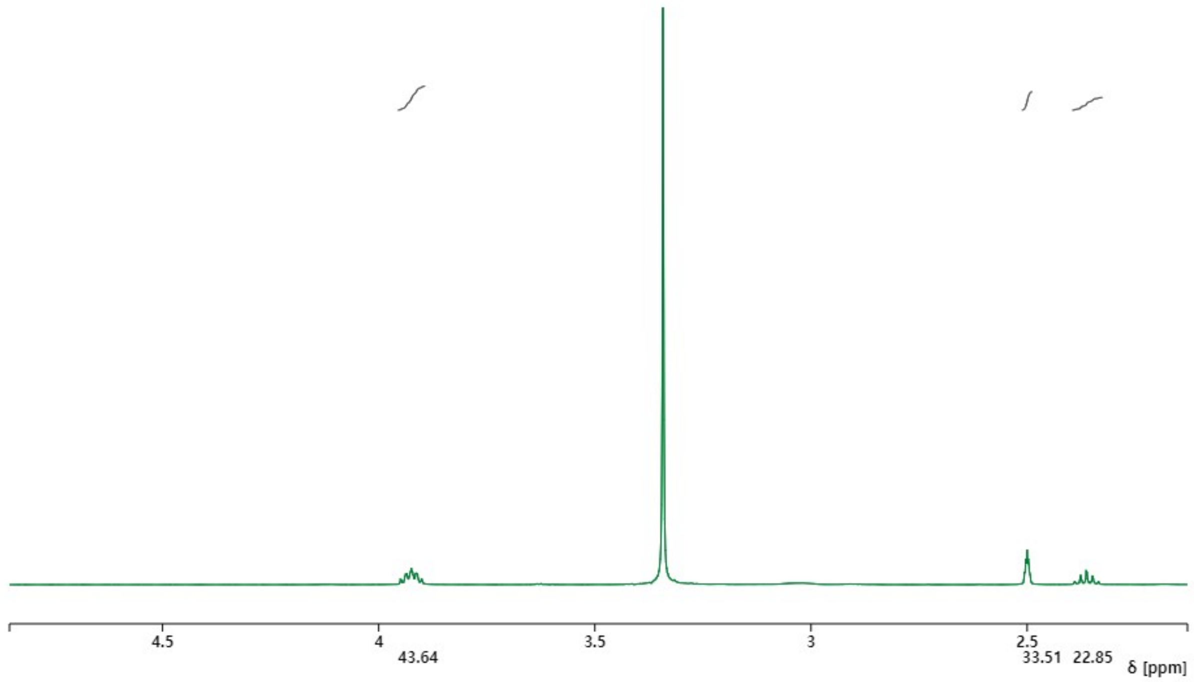

Fig. S1. ^{1}H NMR spectra of AzBr (in dimethyl sulfoxide-d_6). It shows peaks at δ: 2.3-2.4 (2H, NH_2), δ:2.5 (2H, CH_2), δ:3.8-3.9 (4H, 2(CH_2)). The peak at around 3.3 ppm originates from water in the solvent.

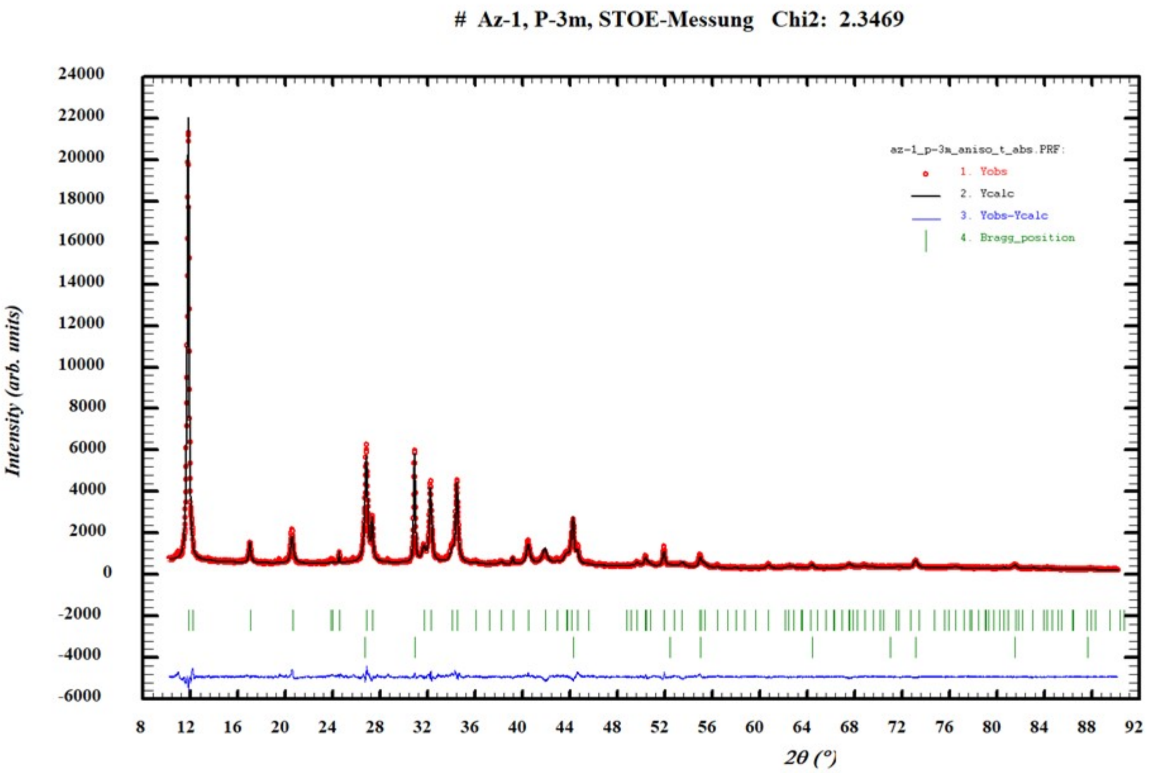

Fig. S2. Final Rietveld plot from polycrystalline powder of $(Az)_2AgBiBr_6$.

Atom	x	y	z	occ.	U11	U22	U33	U12	U13	U23	U(iso)
Br1	0.153(1)	0.305(1)	0.744(2)	1.000							0.084(3)
Ag1	0 000	0.000	0.000	0.68(2)							0.13(3)
Bi1	0.000	0.000	0.000	0.33(2)	0.11(2)	0.11(2)	-0.015(7)	-0.06(2)	0	0	
Ag2	0.000	0.000	0.500	0.33(2)							0.13(3)
Bi2	0.000	0.000	0.500	0.68(2)	0.11(2)	0.11(2)	-0.015(7)	-0.06(2)	0	0	
C1	0.721(1)	0.279(1)	0.879(3)	0.806							0.06(2)
C2	0.611(1)	0.389(1)	0.879(3)	0.806							0.06(2)

Table. S1 Atomic coordinates of the $(Az)_2AgBiBr_6$. The azetidinium cation was simulated by two carbon sites. The occupancy factors of the two carbon sites were fixed and chosen such to represent the complete scattering power of the disordered cation (3 C,1 N, 8 H).

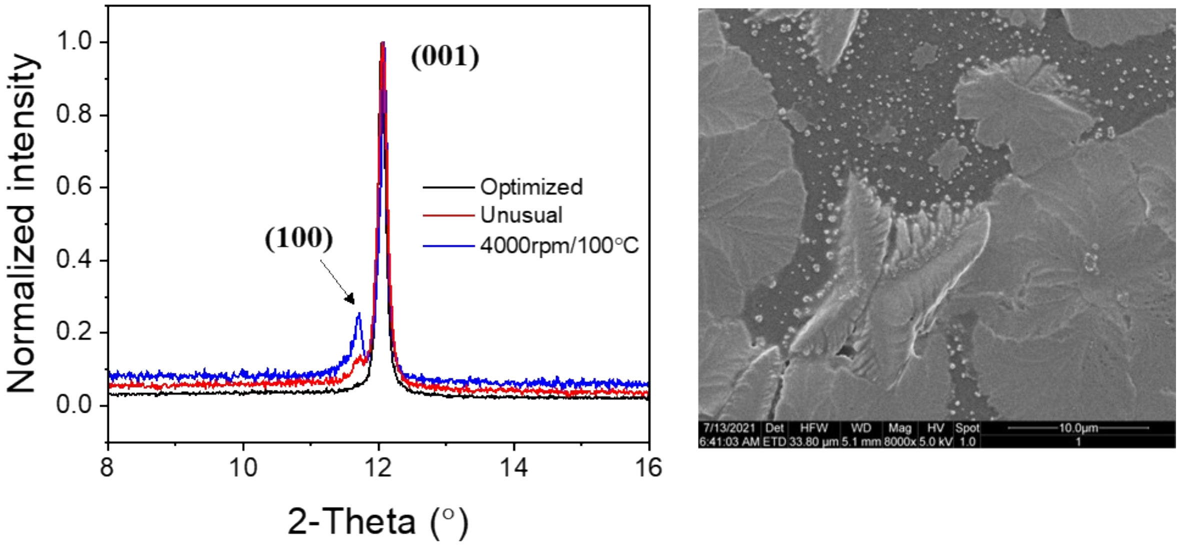

Fig. S3. The (100) peak occurrence of $(Az)_2AgBiBr_6$ thin film compared to unusual spectra from extra optimized sample, and the one from different condition (4000rpm/100°C) (left) with its topographic SEM image (right).

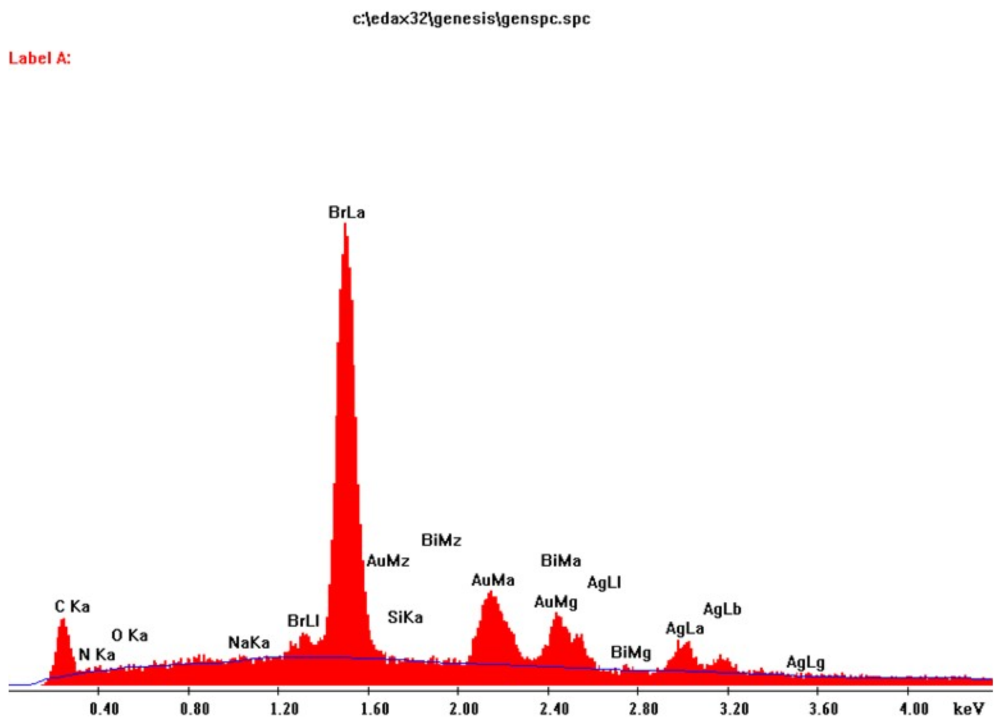

c:\edax32\genesis\genspc.spc
Label :
Acquisition Time : 20:52:11 Date:24-Oct-2023

kV : 15.00 Tilt: 0.00 Take-off:36.38 AmpT : 35.0
Detector Type:SUTW, Sapphire Resolution:135.10 Lsec:39

EDAX ZAF Quantification (Standardless)
Element Normalized
SEC Table : Default

Element	Wt %	At %	K-Ratio	Z	A	F
N K	2.02	13.65	0.0044	1.3446	0.1640	1.0001
O K	0.00	0.00	0.0000	1.3311	0.1910	1.0003
NaK	0.24	1.00	0.0014	1.2378	0.4532	1.0041
BrL	49.99	59.29	0.4255	1.0422	0.8165	1.0003
SiK	0.00	0.00	0.0000	1.3278	0.5007	1.0005
AuM	22.44	10.80	0.1549	0.8937	0.7721	1.0000
BiM	16.40	7.44	0.1133	0.8933	0.7734	1.0000
AgL	8.91	7.83	0.0689	1.0111	0.7644	1.0000
Total	100.00	100.00				

Element	Net Inte.	Bkgd Inte.	Inte. Error	P/B
N K	2.85	10.08	26.71	0.28
O K	0.00	11.80	0.00	0.00
NaK	2.05	21.82	52.51	0.09
BrL	315.47	23.69	0.96	13.31
SiK	0.00	22.05	0.00	0.00
AuM	62.59	19.65	2.56	3.19
BiM	43.47	18.79	3.29	2.31
AgL	25.87	13.97	4.51	1.85

Fig. S4. EDX spectra of $(Az)_2AgBiBr_6$ thin film on glass substrate.

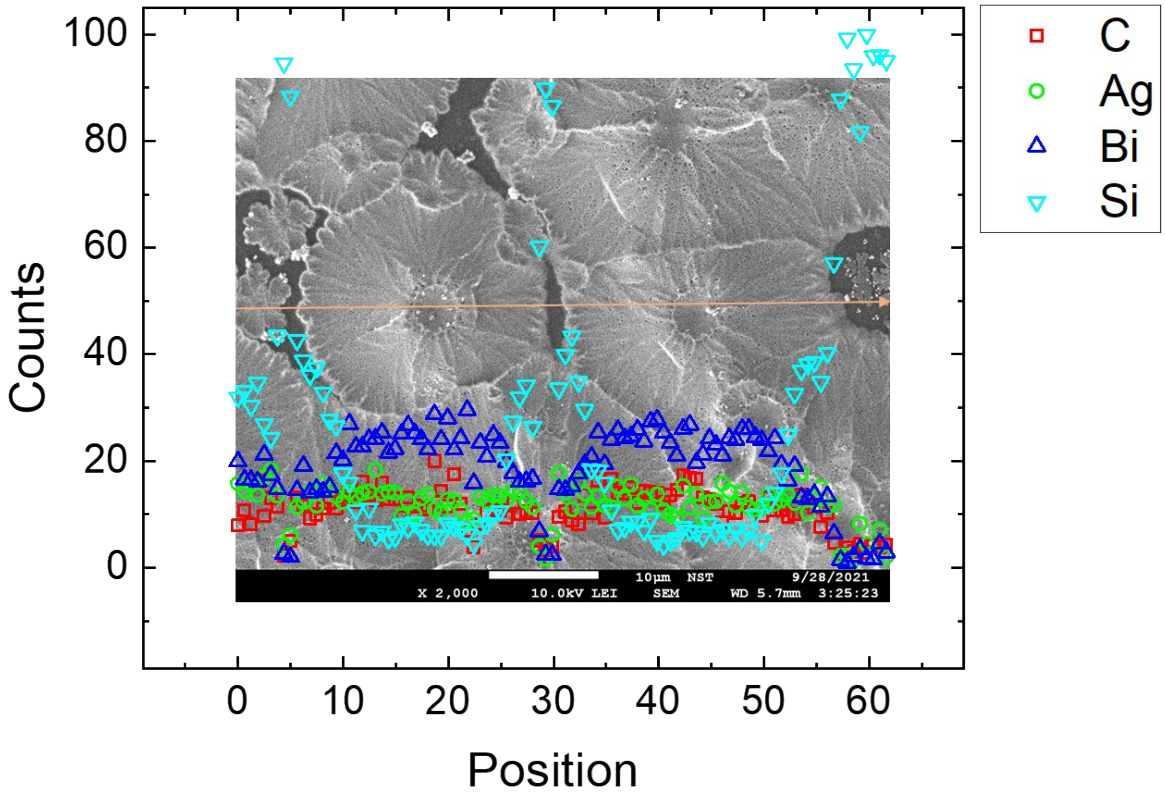

Fig. S5. EDX spectra of $(Az)_2AgBiBr_6$ thin film through linear EDX measurement for the element investigation of the center of the grains (This film was carbon-coated to measure SEM/EDX.).

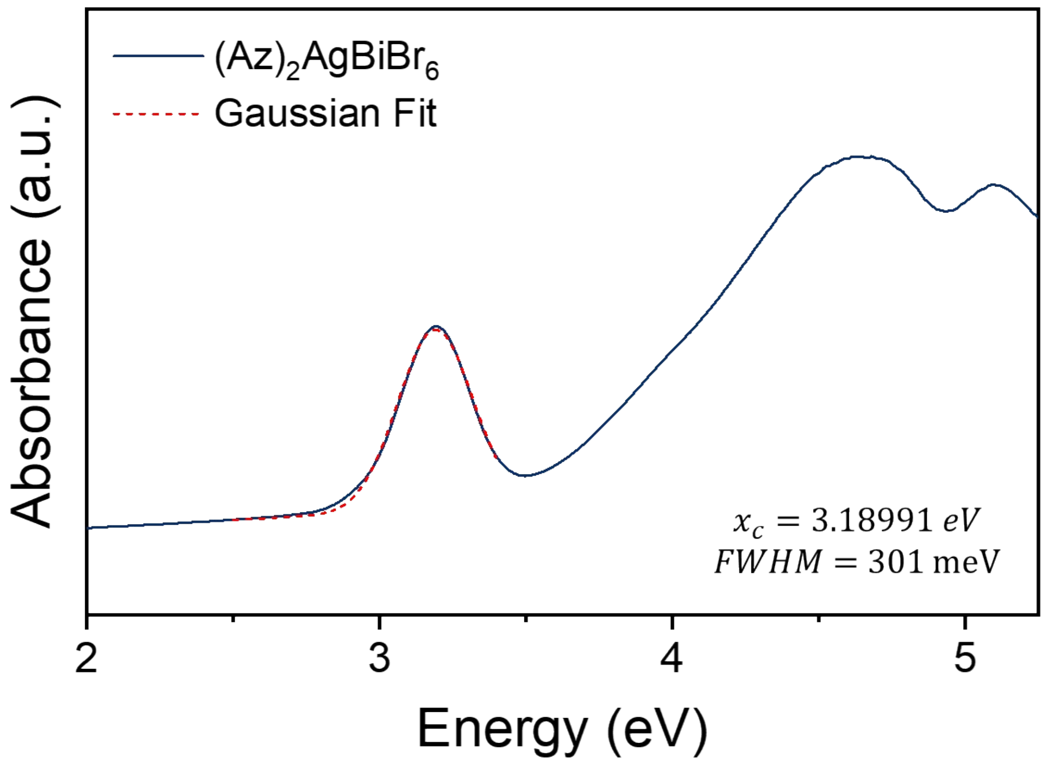

Fig. S6. Description of Gaussian fit with a full-width-at-half-maximum (FWHM) of 301 meV indicating the dominant absorption peak at 3.188 eV in absorbance spectra of $(Az)_2AgBiBr_6$.

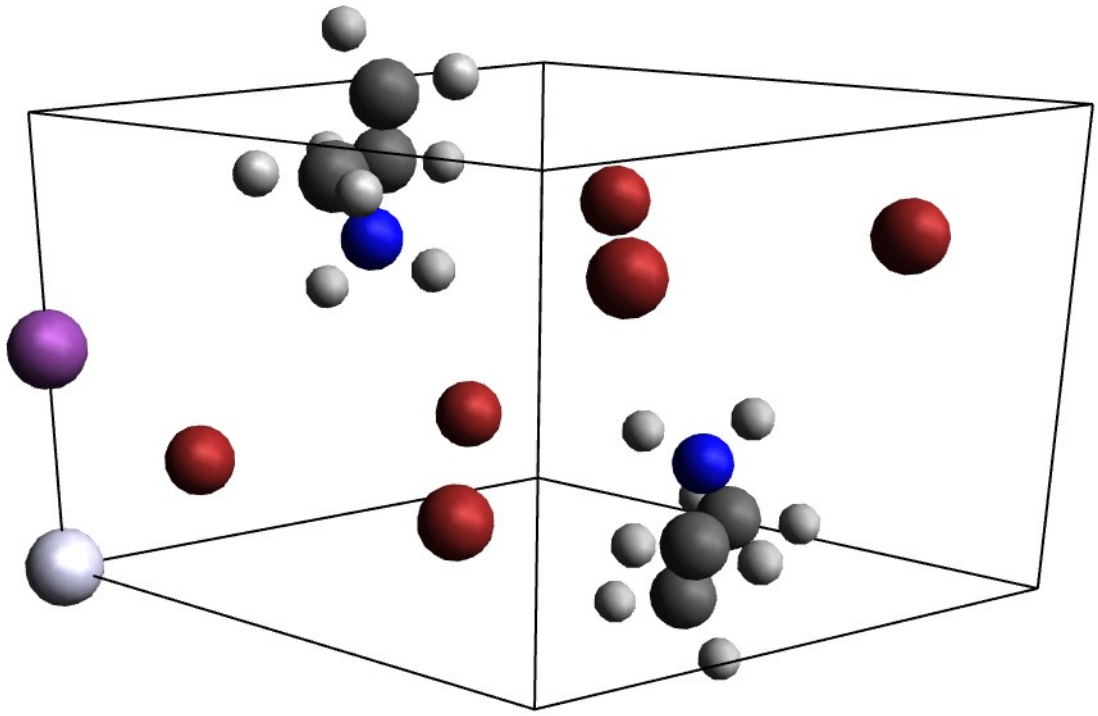

Fig. S7. Unit cell of $(Az)_2AgBiBr_6$, crystal symmetry $P\overline{3}m1$. Here, we have taken the liberty of orienting the Azetidinium cation such that the electron-deficient nitrogen (Blue) part of the Azetidinium cation lies close to the electron-rich bromide (Red) neighbours. The purple sphere denotes the bismuth ion, while the silver sphere denotes the silver ion.

".Strc file"

```
"!STRUCTURE
  !GENERIC LUNIT[AA]=1. !END
  !OCCUPATIONS EMPTY=60 !END
  !KPOINTS R=40. !END
  !LATTICE T= 8.61900 0.00000 0.00000
        -4.30950 7.46427 0.00000
         0.00000 0.00000 7.24160 !END
  !SPECIES NAME='BR' ID='BR_.75_6.0' NPRO=2 2 2 LRHOX=4 !END
  !SPECIES NAME='AG' ID='AG_.75_6.0' NPRO=2 2 2 LRHOX=4 !END
  !SPECIES NAME='BI' ID='BI_.75_6.0' NPRO=2 2 2 2 LRHOX=4 !END
  !SPECIES NAME='N_' ID='N_.75_6.0' NPRO=2 2 2 LRHOX=4 !END
  !SPECIES NAME='C_' ID='C_.75_6.0' NPRO=2 2 2 LRHOX=4 !END
  !SPECIES NAME='H_' ID='H_.75_6.0' NPRO=2 0 0 LRHOX=4 !END

  !ATOM NAME='BR1' R= 0.00000 2.27660 5.38992 !END
```

!ATOM NAME='BR2' R= 4.30950 5.18767 1.85168 !END

!ATOM NAME='BR3' R= 2.33790 6.32597 5.38992 !END

!ATOM NAME='BR4' R= 1.97160 1.13830 1.85168 !END

!ATOM NAME='BR5' R= -2.33790 6.32597 5.38992 !END

!ATOM NAME='BR6' R= 6.64740 1.13830 1.85168 !END

!ATOM NAME='AG1' R= -4.30950 7.46427 7.24160 !END

!ATOM NAME='BI1' R= -4.30950 7.46427 3.62080 !END

!ATOM NAME='N_1' R= 0.00000 4.97618 1.87188 !END

!ATOM NAME='C_1' R= -0.13467 6.01813 0.84040 !END

!ATOM NAME='C_2' R= -0.15224 3.93663 0.84054 !END

!ATOM NAME='C_3' R= 0.28691 4.97379 -0.19824 !END

!ATOM NAME='H_1' R= 0.92100 4.96850 2.32942 !END

!ATOM NAME='H_2' R= -0.74020 4.98259 2.58376 !END

!ATOM NAME='H_3' R= 0.54747 6.86042 0.97558 !END

!ATOM NAME='H_4' R= -1.16464 6.37748 0.74636 !END

!ATOM NAME='H_5' R= -1.18823 3.59478 0.74647 !END

!ATOM NAME='H_6' R= 0.51560 3.08312 0.97562 !END

!ATOM NAME='H_7' R= -0.27594 4.97847 -1.13460 !END

!ATOM NAME='H_8' R= 1.36060 4.96473 -0.41492 !END

!ATOM NAME='N_2' R= 4.30950 2.48809 5.36973 !END

!ATOM NAME='C_4' R= 4.44417 3.53004 6.40121 !END

!ATOM NAME='C_5' R= 4.46174 1.44854 6.40107 !END

!ATOM NAME='C_6' R= 4.02259 2.48570 7.43985 !END

!ATOM NAME='H_9' R= 3.38850 2.48041 4.91219 !END

!ATOM NAME='H_10' R= 5.04970 2.49450 4.65785 !END

!ATOM NAME='H_11' R= 3.76203 4.37233 6.26603 !END

!ATOM NAME='H_12' R= 5.47414 3.88939 6.49525 !END

!ATOM NAME='H_13' R= 5.49773 1.10669 6.49514 !END

!ATOM NAME='H_14' R= 3.79390 0.59503 6.26599 !END

!ATOM NAME='H_15' R= 4.58544 2.49038 8.37621 !END

!ATOM NAME='H_16' R= 2.94890 2.47664 7.65653 !END

```
  !GROUP NAME='MAIN' ATOMS= 'BR1' 'BR2' 'BR3' 'BR4' 'BR5' 'BR6' 'AG1' 'BI1'
    !END
  !GROUP NAME='AZETIDINIUM_1' ATOMS='N_1' 'C_1' 'C_2' 'C_3' 'H_1' 'H_2' 'H_3'
    'H_4' 'H_5' 'H_6' 'H_7' 'H_8' !END
  !GROUP NAME='AZETIDINIUM_2' ATOMS='N_2' 'C_4' 'C_5' 'C_6' 'H_9' 'H_10'
    'H_11' 'H_12' 'H_13' 'H_14' 'H_15' 'H_8' !END
  !CONSTRAINTS
    !RIGID GROUP='AZETIDINIUM_1' !END
    !RIGID GROUP='AZETIDINIUM_2' !END
    !TRANSLATION GROUP='AZETIDINIUM_1' !END
    !TRANSLATION GROUP='AZETIDINIUM_2' !END
    !FREEZE GROUP='MAIN' !END
  !END
!END
!EOB
"
```

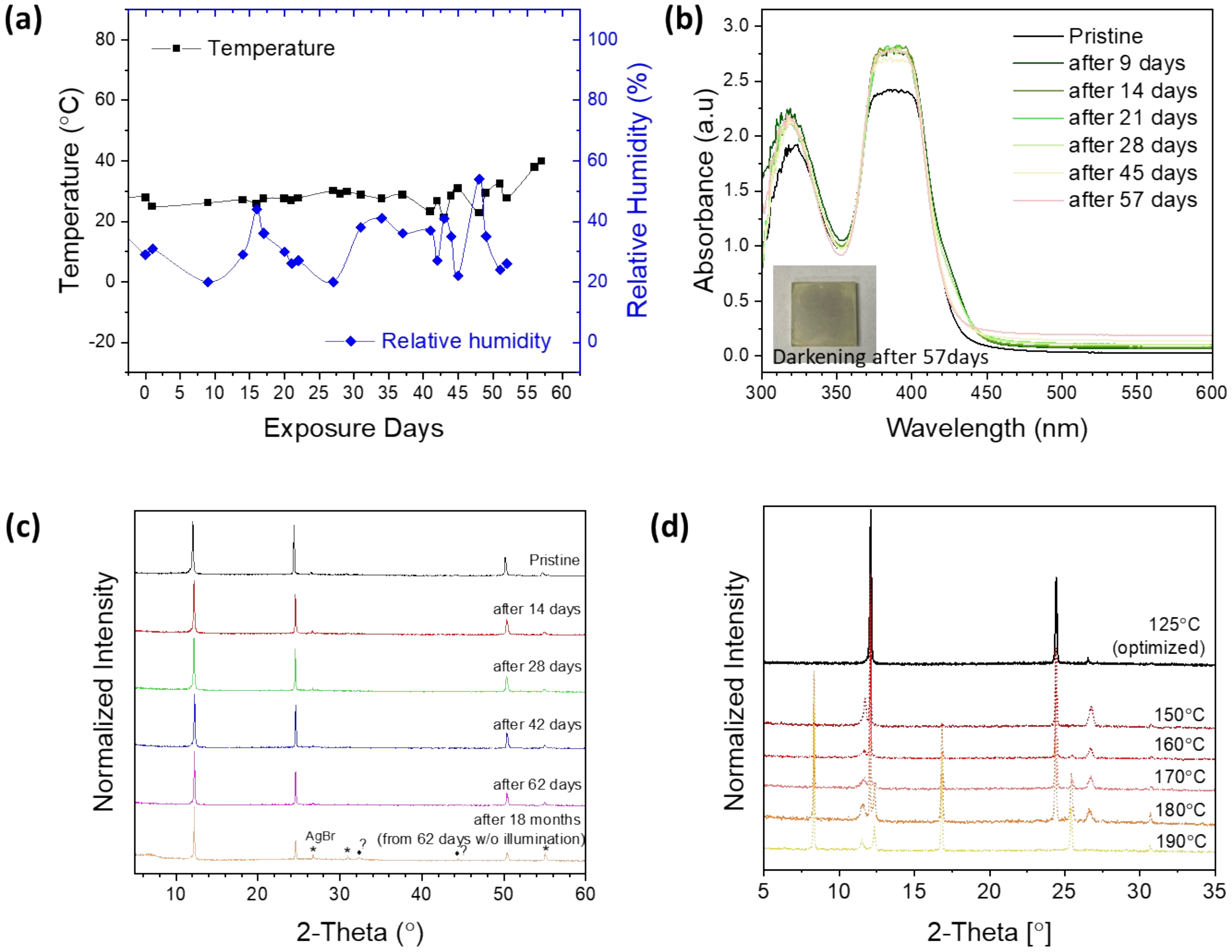

Fig. S8. (a) Record of temperature and humidity over 50 days. (b) Air and light stability test of thin films of $(Az)_2AgBiBr_6$ examined using UV-Vis absorbance measurement during 57 days exposure in ambient and (c) thin film XRD pattern comparison of $(Az)_2AgBiBr_6$ for each day of measurement. (d) Thermal stability test of thin films of $(Az)_2AgBiBr_6$ on a hot plate in a N_2 filled glove box by XRD measurement

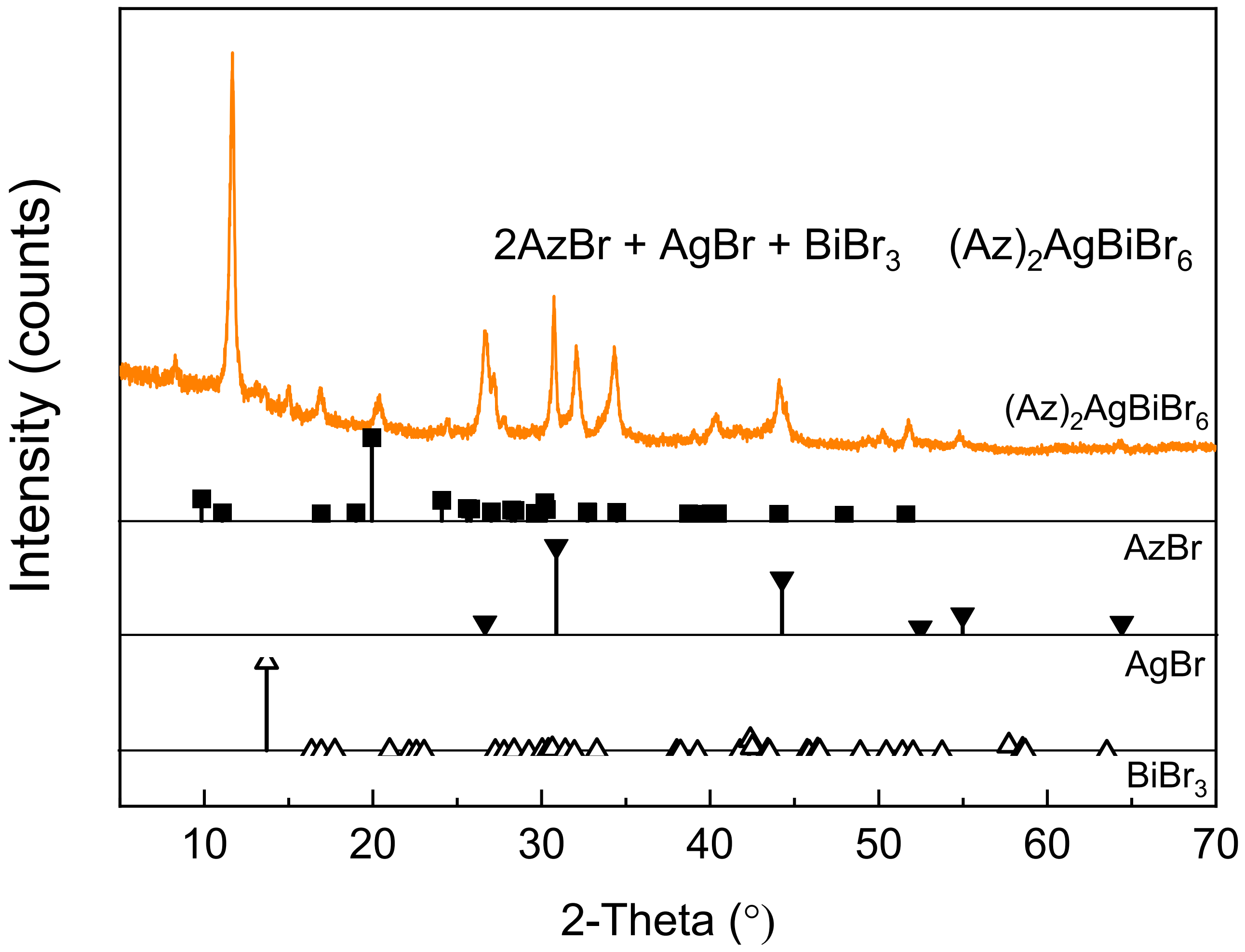

Fig. S9. Powder XRD reflection dataset of optimized $(Az)_2AgBiBr_6$, synthesized AzBr, AgBr, and $BiBr_3$ for the comparison of the reflection patterns.
